# Essential Oils of Five *Baccharis* Species: Investigations on the Chemical Composition and Biological Activities

**DOI:** 10.3390/molecules23102620

**Published:** 2018-10-12

**Authors:** Jane M. Budel, Mei Wang, Vijayasankar Raman, Jianping Zhao, Shabana I. Khan, Junaid U. Rehman, Natascha Techen, Babu Tekwani, Luciane M. Monteiro, Gustavo Heiden, Inês J. M. Takeda, Paulo V. Farago, Ikhlas A. Khan

**Affiliations:** 1 Departamento de Ciências Farmacêuticas, Universidade Estadual de Ponta Grossa (UEPG), Ponta Grossa, PR 84030-900, Brasil; lmmonteiro@hotmail.com (L.M.M.); pvfarago@gmail.com (P.V.F.); 2 National Center for Natural Products Research, School of Pharmacy, University of Mississippi, Mississippi, MS 38677, USA; meiwang@olemiss.edu (M.W.); vraman@olemiss.edu (V.R.); jianping@olemiss.edu (J.Z.); skhan@olemiss.edu (S.I.K.); jurehman@olemiss.edu (J.U.R.); ntechen@olemiss.edu (N.T.); btekwani@olemiss.edu (B.T.); ikhan@olemiss.edu (I.A.K.); 3 Embrapa Clima Temperado, Pelotas, RS 70770-901, Brazil; gustavo.heiden@embrapa.br; 4 Departamento de Meio Ambiente, Universidade Estadual de Maringá (UEM), Umuarama, PR 87020-900, Brazil; takedaines@bol.com.br

**Keywords:** *Baccharis*, antimalarial activity, antitrypanosomal activity, insecticidal activity, GC/MS, DNA barcoding, microscopy

## Abstract

This paper provides a comparative account of the essential oil chemical composition and biological activities of five Brazilian species of *Baccharis* (Asteraceae), namely *B. microdonta*, *B. pauciflosculosa*, *B. punctulata*, *B. reticularioides*, and *B. sphenophylla*. The chemical compositions of three species (*B. pauciflosculosa*, *B. reticularioides*, and *B. sphenophylla*) are reported for the first time. Analyses by GC/MS showed notable differences in the essential oil compositions of the five species. α-Pinene was observed in the highest concentration (24.50%) in *B. reticularioides*. Other major compounds included α-bisabolol (23.63%) in *B. punctulata*, spathulenol (24.74%) and kongol (22.22%) in *B. microdonta*, β-pinene (18.33%) and limonene (18.77%) in *B. pauciflosculosa*, and β-pinene (15.24%), limonene (14.33%), and spathulenol (13.15%) in *B. sphenophylla*. In vitro analyses for antimalarial, antitrypanosomal, and insecticidal activities were conducted for all of the species. *B. microdonta* and *B. reticularioides* showed good antitrypanosomal activities; *B. sphenophylla* showed insecticidal activities in fumigation bioassay against bed bugs; and *B. pauciflosculosa*, *B. reticularioides*, and *B. sphenophylla* exhibited moderate antimalarial activities. *B. microdonta* and *B. punctulata* showed cytotoxicity. The leaves and stems of all five species showed glandular trichomes and ducts as secretory structures. DNA barcoding successfully determined the main DNA sequences of the investigated species and enabled authenticating them.

## 1. Introduction

*Baccharis* L. (Asteraceae) is an important genus comprising 435 species distributed from Argentina to the United States (USA). In Brazil, the genus is represented by 179 species [1]. Several species of *Baccharis* are frequently used in traditional medicine as analgesic, antidiabetic, anti-inflammatory, digestive, diuretic, and spasmolytic agents [2,3]. These properties provide an excellent rationale for systematically studying their therapeutic properties. However, the correct identification of different *Baccharis* species is challenging due to their morphological similarities. Moreover, many different species of *Baccharis* are collectively called “vassouras” in Brazil, causing further confusion [4].

*Baccharis* species have also provided valuable biomolecules in the discovery of new medicinal natural products [5]. Species of the genus produce essential oils (EOs) that are composed mainly of monoterpenoids and sesquiterpenoids. Several such EOs are used in the fragrance industry and for pharmaceutical purposes [3,6,7,8].

Many of the medicinal properties described for *Baccharis* are attributed to their EOs [3]. The EOs of the genus have been reported to have several biological activities, including antibacterial, antifungal [9,10], antiprotozoal, antiviral, antioxidant, anti-inflammatory, antimutagenic, antiulcer, chemopreventive, repellent, sedative [7,11,12,13], schistosomicidal [14], and larvicidal against *Aedes aegypti* [15] and cytotoxic properties [16]. *B. microdonta* DC. has shown antibacterial activity against *Salmonella typhi* [17] and anti-inflammatory properties [18]. Another species, *B. pauciflosculosa* DC., has exhibited antimicrobial activities [17,19]. 

However, few studies have been carried out on the chemical analysis of the EOs of *Baccharis* species [20]. Thus, the major aims of this study were to identify the marker compounds and compare the chemical profiles of the EOs of five *Baccharis* species, namely *B. microdonta*, *B. pauciflosculosa*, *B. punctulata* DC., *B. reticularioides* Deble and A.S.Oliveira, and *B. sphenophylla* Dusén ex Malme, collected from Brazil. To the best of our knowledge, no previous reports exist on the antitrypanosomal, antimalarial, or insecticidal activities of these five species. The study also analyzes the micromorphology of secretory structures, in which the EOs are stored by microscopy and discriminate the species by DNA barcoding.

## 2. Results and Discussion

### 2.1. Yield and Chemical Composition of Essential Oils

Essential oils are liquid, volatile, clear, and rarely colored substances that are soluble in organic solvents and usually of lesser density than water. EOs extracted by hydrodistillation from the vegetative aerial parts of the *Baccharis* species presented a strong and characteristic aroma. The EO of *B. punctulata* was green, whereas it was yellow in *B. microdonta* and light yellow in the other species. The yield of EO was 0.58% (*v*/*w*) in *B. microdonta*, 0.93% (*v*/*w*) in *B. pauciflosculosa*, 0.29% (*v*/*w*) in *B. punctulata*, 0.59% (*v*/*w*) in *B. reticularioides*, and 0.53% (*v*/*w*) in *B. sphenophylla*. As per the literature, the yield of EOs in the *Baccharis* species ranged between 0.08–2.82%. *B. obovata* Hook. and Arn. presented the highest yield [21], whereas the lowest content was achieved for *B. lateralis* Baker (syn. *B. schultzii* Baker) [22], which were collected in Argentina and Brazil, respectively.

The yield of EOs can be influenced by the physiological variations inherent in the plant, environmental conditions, phenological factors, genetic characteristics of the cultivars, drying conditions applied to the plant material prior to extraction, the process employed for grinding, the storage conditions, and the EO extraction methods [23,24,25]. Different yields of EOs extracted from the leaves of *B. microdonta* collected in São Paulo, Brazil were reported. Sayuri et al. [26] showed yields between 0.06–0.35%, whereas Lago et al. [22] presented yields of 0.08–0.21% for *B. microdonta*. However, these authors extracted the EOs only from the leaves of *B. microdonta*. In the present work, mixtures of leaves and stems were used for extraction. The anatomical study of *Baccharis* species revealed several large secretory cavities in the cortex of the stems, which may have contributed to the increased yield, as observed by Saulle et al. [27] for *Eucalyptus saligna* Sm. (Myrtaceae).

Chemically, EOs are complex mixtures that can contain 20–60 compounds in different concentrations, and are characterized by two or three main components with higher concentrations compared to others present in lower concentrations. In general, the major compounds that are present in EOs are responsible for the biological activities [28,29].

**Figure 1:**
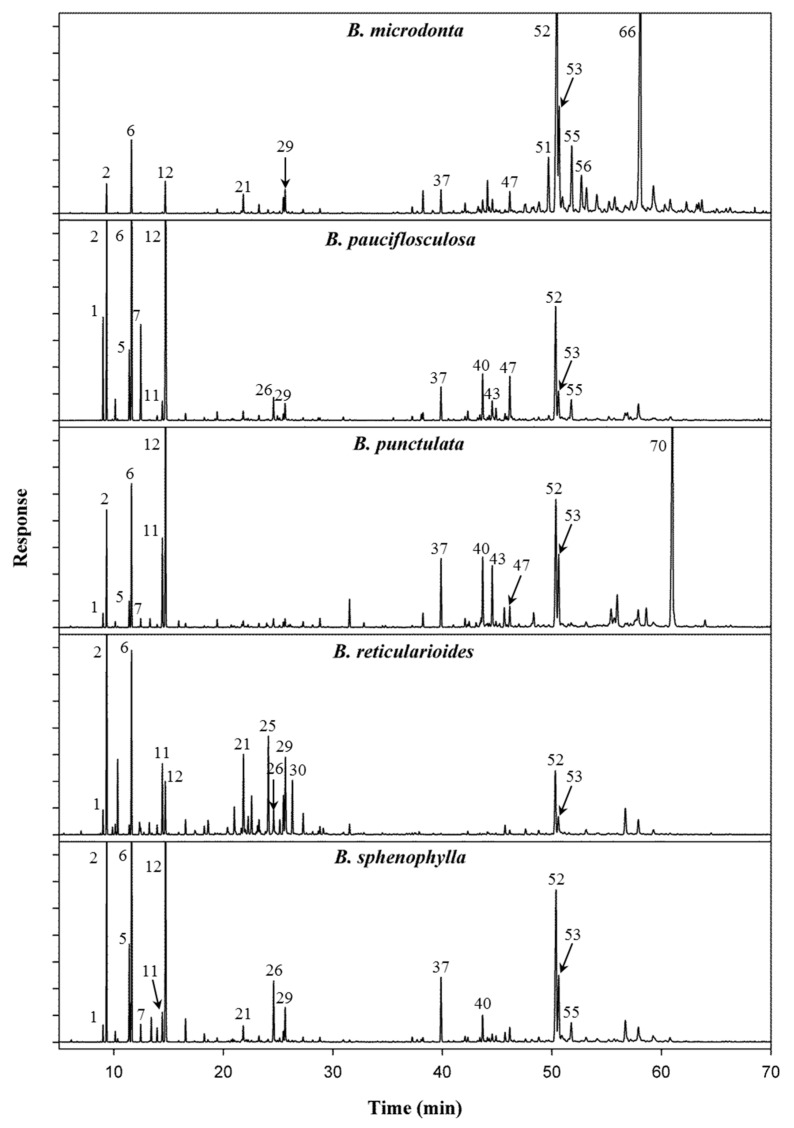
GC chromatograms of *Baccharis* species obtained for non-polar column. Compound identification is consistent with Table 1.

The chemical compositions of EOs from the five species of *Baccharis* were investigated, and their profiles (Figure 1) were compared. Table 1 compares relative retention indices (RRIs) using non-polar and polar columns, the literature-reported retention indices (RI lit), chemical identity, and relative peak area percentage (%) concentration of the chemical constituents of the five species. 

Monoterpenoids and sesquiterpenoids are frequently found in the EOs of *Baccharis* [3,30]. Sesquiterpenoids seemed to be more abundant in the majority of the species [3,6,7,16,22,30,31,32,33]. However, the EO of some species contained more monoterpenoids than sesquiterpenoids, such as in *B. obovata* [21], *B. schultzii*, *B. regnelli* Sch. Bip. ex Baker, *B. uncinella* DC. [22], *B. darwinii* Hook. & Arn. [34], *B. tridentata* Vahl. [33] and *B. trimera* (Less.) DC. [35] 

Considering that raw percentages of volatile compounds are usually reported by the use of the non-polar GC column, in the present work, only these percentages were discussed. Both monoterpenoids and sesquiterpenoids were found in the EOs of all of the *Baccharis* species that were analyzed, although in different concentrations. Higher concentrations of monoterpenoids (60.78%) were found in *B. reticularioides*, which was formed by 27.45% of monoterpenoids hydrocarbons and 33.33% of oxygenated monoterpenoids. In the case of sesquiterpenoids, *B. microdonta* showed the highest concentrations (41.66%), comprising 18.33% of sesquiterpenoids hydrocarbons and 23.33% of oxygenated sesquiterpenoids. The sesquiterpenoid cyclic alcohols found in the present study, such as ledol, spathulenol, viridiflorol, and palustrol, are not only important in the perfume industry due to their agreeable aromatic notes, but also have taxonomic value [33].

A recent bibliographic review reported around 60 compounds identified in the EOs of 16 species of *Baccharis* that showed biological activities. The main components that were found in these species were α-thujene, β-caryophyllene, β-pinene, camphor, caryophyllene, caryophyllene oxide, limonene, nerolidol, thymol, thymol acetate, thymol methyl ether, sabinene, and spathulenol [3]. In the present study, α-pinene, β-pinene, limonene, trans-pinocarveol, pinocarvone, myrtenal, α-terpineol, cis-carveol, carvone, β-selinene, δ-cadinene, spathulenol, caryophyllene oxide, and δ-cadinol were found in all of the species of *Baccharis* studied. However, significant differences in their concentrations were observed.

The compounds’ identification is given in Table 1. *B. microdonta* contained spathulenol (22.74%) (Figure S2) and kongol (22.22%) (Figure S1), *B. pauciflosculosa* showed β-pinene (18.33%) and limonene (18.77%), *B. punctulata* contained α-bisabolol (23.63%), *B. reticularioides* presented α-pinene (24.50%), and *B. sphenophylla* showed α-pinene (10.74%), β-pinene (15.24%), limonene (14.33%), and spathulenol (13.15%) as the major compounds. It is important to highlight that kongol and α-bisabolol were found only in *B. microdonta* and *B. punctulata*, respectively. These compounds can be considered chemical markers for these species. 

Differences in the EO chemical compositions have been reported for *B. punctulata* collected from different geographical locations. The EOs of the samples collected from Uruguay have shown β-phellandrene (5.2%), bornyl acetate (5.2%), α-cadinol (4.2%), δ-elemene (3.7%), and the ketone shyobunone (3.5%) as the major compounds [36], whereas the EOs sourced in Guaíba, Brazil have comprised bicyclogermacrene (9.73%), cis-cadin-4-en-7-ol (6.77%), and (Z)-ocimene (6.33%) [23]. Of these compounds, only bornyl acetate (1.32%) and bicyclogermacrene (3.10%) were found in the present study in *B. punctulata* and in low concentrations. α-Bisabolol was not reported in the previous studies, but it was found in higher concentration (23.63%) in the present work. 

For *B. microdonta*, Lago et al. [22] reported 24% of caryophyllene oxide, whereas Sayuri et al. recorded 31% of elemol, 34% of spathulenol, 19% of β-caryophyllene, and 24% of germacrene D as the major compounds. Both of these studies used samples collected from Campos do Jordão, Brazil. None of the earlier studies have reported kongol from any of the five *Baccharis* species, whereas this compound was found in high quantity (22.22%) in *B. microdonta* in the present study. Even though the chemical composition of EOs is frequently associated to environmental and phenological influences, it is necessary to investigate whether these variations in *B. punctulata* and *B. microdonta* are possibly linked to different chemotypes. Not only the compositions of EOs, but also the quantities of the compounds vary throughout the life of the plant. This is related to the circadian rhythms, seasonal conditions, and environmental influences that impact the development of the species [24]. To avoid some of these factors, in the present work, all five species were grown in the same locality and collected on the same day and at the same time. Additionally, the sample preparation, hydrodistillation, and characterization of the EOs by GC/MS analysis of all of the materials were performed under the same experimental conditions.

Retta et al. [8] analyzed five species of *Baccharis*, namely *B. gaudichaudiana* DC., *B. microcephala* (Less.) DC., *B. penningtonii* Heering, *B. phyteumoides* (Less.) DC., and *B. spicata* (Lam.) Baill., and reported that they were qualitatively, but not quantitatively, similar. In the present study, although the five species presented similar qualitative patterns, some compounds were found only in one specific species. Qualitative similarities in these species were expected, as they belonged to the same genus. However, they were classified into two different taxonomic groups: *B. punctulata* belonged to subgenus *Molina*, whereas the other four species belonged to the subgenus *Baccharis*.

By comparing the GC chromatograms of the EOs of the five species of *Baccharis*, it is possible to distinguish them by the quality and quantity of their major constituents (Figure 1).

### 2.2. Antimalarial Activity

In order to explore the antimalarial properties of the five species of *Baccharis*, their EOs were investigated against chloroquine-sensitive (D6) and chloroquine-resistant (W2) strains of *Plasmodium falciparum* (Table 2). The EOs of *B. microdonta* and *B. punctulata* were cytotoxic to Vero cells (selectivity control), and because of this result, they were not indicated to be used in cellular media as an antimalarial. These two EOs differed from other studied species due to the presence of the chemical markers spathulenol (22.74%) and kongol (22.22%) for *B. microdonta* and α-bisabolol for *B. punctulata* (23.63%). Due to their cytotoxic properties, these EOs can be further explored in other cytotoxicity or anticancer studies, as previously reported by Pereira et al. for *B. milleflora* DC. [16]. Otherwise, the EO of *B. pauciflosculosa* showed moderate antimalarial activity against both *P. falciparum* clones (lower than 15 μg/mL), while *B. reticularioides* and *B. sphenophylla* demonstrated discrete antimalarial effects. Significant differences in the quality and quantity of the chemical components of the five EOs can be strongly related to these data. In particular, the variation in the quantities of the main components e.g., monoterpenes β-pinene (18.33%) and limonene (18.77%), might be responsible for the antimalarial effect of the EO of *B. pauciflosculosa*.

In the genus *Baccharis*, antimalarial studies were carried out for a few species using their plant extracts or isolated compounds. *B. dracunculifolia* DC. is the most important plant source of the Brazilian green propolis, and showed antimalarial activities against *P. falciparum* (D6) using crude hydroalcoholic green propolis extract (13 μg/mL) and hautriwaic acid lactone with IC_50_ values of 0.8 µg/mL (D6 clone) and 2.2 µg/mL (W2 clone) [39]. The extracts of leaves from *B. rufescens* Spreng. and *B. genistelloides* (Lam.) Pers. also showed in vitro antimalarial activity, achieving 100% of inhibition at 100 μg/mL against a *P. falciparum* chloroquine-resistant strain [40]. In spite of the antiplasmodial activity of plant EOs widely reported in the literature [28], the present work represents the first study involving the antimalarial effect using the EOs of the *Baccharis* species.

Additionally, the selectivity index (SI) was calculated to predict how toxic the samples were to normal cells. The calculated selectivity indices showed that *B. pauciflosculosa* had better selectivity to *P. falciparum* clones than for Vero cells. This EO was safer than other examined samples, making it a candidate for further development as an antimalarial agent, mainly via the inhalation route.

### 2.3. Antitrypanosomal Activity

*Trypanosoma brucei* is a protozoan that causes human African trypanosomiasis (HAT). There are currently only four drugs available for its treatment, namely pentamidine, melarsoprol, suramin, and eflornithine. Considering the lack of phytochemical and pharmacological data available for plants with efficacy against trypanosomes and aiming at proposing alternative treatments for HAT, an initial screening of the EOs of the five species of *Baccharis* were carried out against *T. brucei* (Table 3).

All five species presented remarkable antitrypanosomal activities at concentrations ranging from 0.31–1.69 µg/mL (IC_50_) and 0.52–2.68 µg/mL (IC_90_). *B. pauciflosculosa* demonstrated the highest effect, 0.31 µg/mL (IC_50_) and 0.52 µg/mL (IC_90_), followed by *B. reticularioides*, which showed 0.96 µg/mL (IC_50_) and 2.49 µg/mL (IC_90_), and *B. sphenophylla*, which presented 1.14 µg/mL (IC_50_) and 2.38 µg/mL (IC_90_). This is the first report on the antitrypanosomal activities for these species. Good EO activity was also reported for other species, such as *Cymbopogon giganteus* Chiov. (Poaceae) (IC_50_ of 0.25 μg/mL) [41] and *Juniperus oxycedrus* L. (Cupressaceae) (IC_50_ of 0.9 μg/mL) [42].

Costa et al. [42] investigated some isolated monoterpenoids (1,8-cineole, borneol, camphor, carvacrol, citral, eugenol, linalool, thymol, and α-pinene) against *T. brucei*. α-Pinene and citral exhibited the highest activities with IC_50_ values of 2.9 μg/mL and 18.9 μg/mL, respectively. Therefore, the activities observed in this work could be attributed to the presence of α-pinene in the EOs of *B. pauciflosculosa* (10.45%), *B. reticularioides* (24.50%), and *B. sphenophylla* (10.74%). *B. microdonta* and *B. punctulata* demonstrated a lower antitrypanosomal effect than other species, and contained low concentrations of α-pinene (0.72% and 3.55%, respectively).

### 2.4. Insecticidal Studies with Bed Bugs

Most of the plant-based insecticides and repellents are derived from plants containing EOs [43]. In addition, receptors responding to DEET (*N*,*N*-diethyl-3-methylbenzamide) can also respond to volatile terpenes [44]. Therefore, this exploratory study was aimed at investigating the insecticidal potential of EOs of *Baccharis* species against bed bugs because of the increasing demands for information about effective control tactics and their public health risks. 

**Figure 2:**
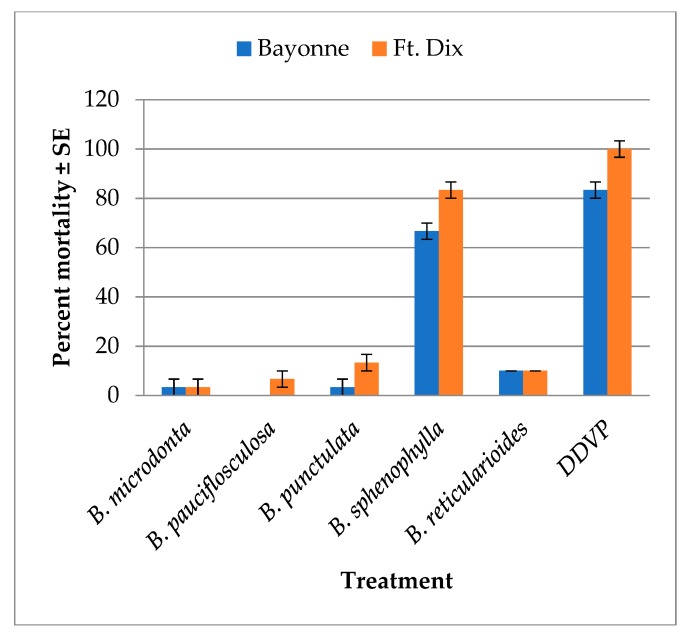
Mean percent mortality (±SE) caused by the essential oils of five species of *Baccharis* against two strains of bed bugs (*Cimex lectularius*) 24 h after treatment in a fumigation bioassay. Essential oil dose: 250 µg/125 mL of air, 2,2-dichlorovinyl dimethyl phosphate (DDVP) used as standard (2 µg/125 mL of air). Mean and standard error were calculated in John´s Macintosh Project (JMP) 10.0.

The results of fumigation studies involving bed bugs are illustrated in Figure 2. Out of the five EOs analyzed, only *B. sphenophylla* produced 66.67 ± 3.33% mortality in the insecticide-resistant strain ‘Bayonne’, while producing 83.33 ± 3.33% mortality in the susceptible strain ‘Ft.Dix’, 24 h after treatment. All of the other EOs showed less than 15% mortality. In particular, *B. sphenophylla* EO exhibited a wide range of volatile compounds with no specific chemical markers. In that sense, its fumigation effect could be attributed to the synergic effects of terpenes, as previously reported in the literature [45]. Several monoterpenes were isolated and demonstrated fumigation effects on different insects, e.g., α-pinene, β-pinene, 3-carene, limonene, myrcene, α-terpinene, and camphene [43]. Of these compounds described in the literature, only camphene was not present in the EO of *B. sphenophylla*, which reinforces the hypothesis that its effect was based on a synergism of various volatile compounds present in the EO.

**Figure 3:**
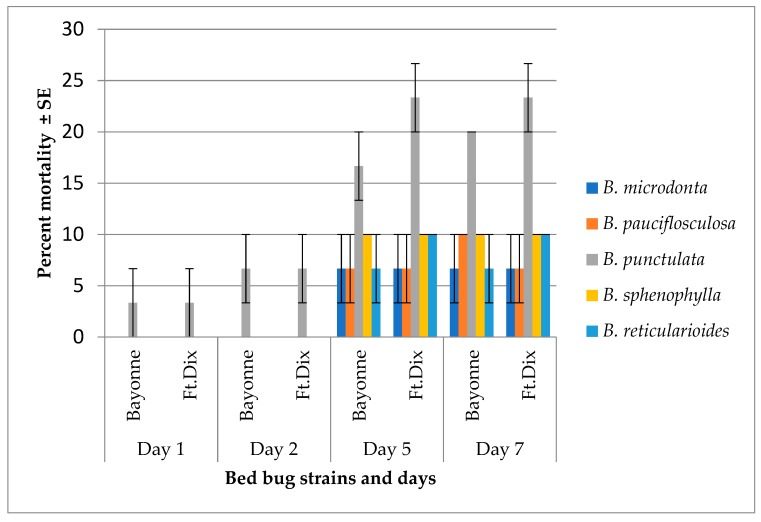
Mean percent mortality (±SE) caused by the essential oils (EOs) of five species of *Baccharis* applied topically on bed bug *Cimex lectularius*. Essential oil dose: 50 µg/bug, deltamethrin (standard) produced 100% (Ft. Dx.) and 56.67% (Bayonne) mortality at 2.4 ng/bug 24 h after treatment. Mean and standard error were calculated in JMP 10.0.

None of the EOs showed high mortality when applied topically at 50 µg/bug. Only in *B. punctulata* did the mortality reach 20% seven days after the treatment (Figure 3). In residual study, none of the EOs produced mortality in bed bugs seven days of exposure at 100 µg/cm^2^. 

### 2.5. Secretory Structures

In Asteraceae, EOs are biosynthesized and accumulated in various secretory structures, such as idioblast oil cells, oil cavities, secretory ducts, and glandular trichomes [46]. In *Baccharis*, EOs can be found in roots, stems, leaves and flowers [35,47,48], and are stored in secretory ducts and glandular trichomes [4]. 

**Figure 4:**
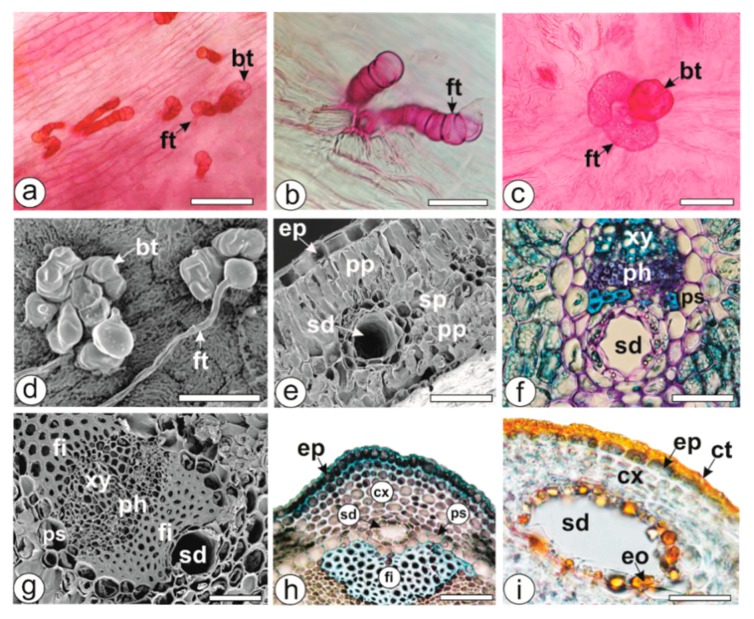
Anatomy of *Baccharis* [light (**a**,**b**,**c**,**f**,**h**,**i**) and scanning electron microscopy (**d**,**e**,**g**)]. Leaf epidermis in surface view (**a**–**d**), cross-sections of the leaf (**e**–**g**) and of the stem (**h**,**i**). *B. microdonta* (**a**), *B. pauciflosculosa* (**b**,**e**,**f**), *B. punctulata* (**c**,**h**), *B. reticularioides* (**d**,**g**), *B. sphenophylla* (**i**). [bt–biseriate glandular trichome, ct—cuticle, cx—cortex, eo—essential oil, ep—epidermis, fi—fibers, ft—flagelliform trichome, ph—phloem, pp—palisade parenchyma, sd—secretory ducts, sp—spongy parenchyma, xy—xylem]. Scale bars: **b**, **c**, **d**, **f**, **g**, *i* = 50 µm; *e* = 100 µm; **a**, *h* = 200 µm.

In the present study, the leaves and stems of all of the *Baccharis* species showed glandular trichomes, either isolated or in clusters (Figure 4a–d), and frequently inserted in small epidermal depressions. There were three types of glandular trichomes, namely biseriate (Figure 4a,c,d), flagelliform with straight body (Figure 4a,b,d), and flagelliform C-shaped (Figure 4c). Biseriate glandular trichomes were present in all of the species, except for *B. pauciflosculosa*. The flagelliform trichomes with straight body were found in all of the species except *B. punctulata*, and only this species had flagelliform C-shaped trichomes (Figure 4c). 

All the studied *Baccharis* species presented secretory ducts in the mesophyll (Figure 4e,f) and midrib of the leaves (Figure 4g), and in the cortex of the stems (Figure 4h,i). They showed a uniseriate epithelium formed by four to 20 cells with large nuclei and dense cytoplasm containing EO droplets, and were found next to the parenchyma sheath near the phloem (Figure 4e–i). These secretory ducts could also sometimes release other chemical compounds such as resins and tannins beside EOs [49]. Essential oils in the trichomes and ducts, and lipophilic compounds in the cuticle reacted positively with Sudan III in the histochemical tests (Figure 4i).

### 2.6. Identification of the Samples by DNA

Classical methods for the identification of medicinal plants include organoleptic, macroscopic, and microscopic methods and chemical profiling. Modern techniques, such as DNA barcoding, have emerged recently and are often used in plant identification [50]. Considering the morphological similarities among *Baccharis* species [4], all four genomic regions, namely ITS, ETS, psbA-trnH, and trnL-trnF were subjected to amplification and sequencing in order to provide molecular data for the differentiation of the species. Only two samples (ECT0000641, ECT0000642) resulted in an ETS PCR product and consequently sequence data. Only a single sequence per authenticated species was available for sequence comparison. Table 4 shows KP2 distances between five *Baccharis* samples and authenticated species. The KP2 value determines the genetic distance between samples and the lowest KP2 value of 0.000 indicates a 100% match. The psbA-trnH sequences of samples *B. reticularioides* (ECT0000642) and *B. sphenophylla* (ECT0000647) matched 100% to three different species, indicating that the genomic region has not much variation in its sequence to be helpful to distinguish between species.

The samples analyzed were morphologically identified. The ITS sequences of samples *B. pauciflosculosa*, *B. reticularioides*, *B. sphenophylla*, and the trnL-trnF sequences of samples *B. microdonta* and *B. punctulata* support the species identification based on morphology as the sequences 100% matched the sequences from previously authenticated samples.

Only single sequences of authenticated samples were available for the sequence alignments. To achieve a more reliable way of sample identification based on genomic regions, more authenticated samples should be analyzed to get a better representation of the intraspecific sequence variations of a certain species.

## 3. Materials and Methods

### 3.1. Plant Material

Fresh samples of vegetative aerial parts were collected, in triplicate, from *B. microdonta*, *B. pauciflosculosa*, *B. punctulata*, *B. reticularioides*, and *B. sphenophylla* in March 2016 from open and sunny habitats in Campos Gerais, Ponta Grossa, Paraná, Southern Brazil (coordinates 25°5′11′′ S and 50°6′23′′ W). The specimens were registered as ECT0000644 (*B. microdonta*), ECT0000641 (*B. pauciflosculosa*), ECT0000645 (*B. punctulata*), ECT0000642 (*B. reticularioides*), and ECT0000647 (*B. sphenophylla*), and deposited in the Herbarium of Embrapa Clima Temperado (ECT) in Rio Grande do Sul, Brazil. The access to the botanical material was authorized and licensed by the Conselho de Gestão do Patrimônio Genético (CGEN/SISGEN) registered under number A429DA6. The collected plant materials were selected and standardized in order to obtain leaves and stems with the same pattern. Then, the materials were dried in the shade at room temperature and cut into small pieces (~1 cm).

### 3.2. Extraction of Essential Oil (EO)

Dried plant material (100 g) was subjected to hydrodistillation for 3 h, in triplicate, using a Clevenger-type apparatus for the extraction of EOs. The EOs obtained were dried using anhydrous Na_2_SO_4_, stored in glass vials with Teflon-sealed caps, and kept under −4 ± 0.5 °C with no light until analysis. The yield of EO was calculated in volume/mass % [51].

### 3.3. Chemicals

GC-grade *n*-hexane (>99%) was purchased from Sigma Aldrich (St. Louis, MO, USA). The reference standards of α-pinene, β-pinene, camphene, sabinene, α-thujone, β-myrcene, *p*-cymene, limonene, γ-terpinene, α-terpineol, terpinolene, *trans*-pinocarveol, terpinen-4-ol, verbenone, β-elemene, carveol, bornyl acetate, caryophyllene oxide, viridiflorol, α-bisabolol, and myrtenal were also purchased from Sigma-Aldrich. 

Kongol (Figure S1) and spathulenol (Figure S2) were isolated from EO of *B. microdonta* and identified by NMR spectroscopy in the present study. Briefly, 180 mg of EO of *B. microdonta* was subjected to a Biotage ZIP KP-SIL 45-g cartridge, and the isolation was performed on a Biotage Isolera^TM^ system (Biotage, Charlotte, NC). Hexanes-ethyl acetate was used for eluting with increasing proportions of ethyl acetate from 0% to 20%. The eluted fractions (12 mL each) were collected and detected by using thin layer chromatography. Kongol (23.8 mg) and spathulenol (25.3 mg) were obtained from the fractions 102–106 and 95–99, respectively. The proton and carbon NMR spectra of the two isolates were recorded using an Agilent DD2-500 NMR spectrometer (Agilent, Santa Clara, CA) equipped with a One NMR probe operating at 499.79 MHz for ^1^H and 125.67 MHz for ^13^C. The spectrum of spathulenol was identical to that of the reference standard. The spectral data of isolated kongol was also in agreement with that reported in the literature [52].

### 3.4. Gas Chromatography-Mass Spectrometry (GC/MS) Analysis

The EOs of *B. microdonta*, *B. pauciflosculosa*, *B. punctulata*, *B. reticularioides,* and *B. sphenophylla* were analyzed by GC/MS using an Agilent 7890A GC system equipped with a 5975C quadrupole mass spectrometer and a 7693 autosampler (Agilent Technologies, Santa Clara, CA, USA). Ten microliters of EOs were dissolved in 1 mL of *n*-hexane for each oil sample, and 1 µL of the sample solution was injected. Helium was used as the carrier gas at a flow rate of 1 mL/min. The inlet temperature was set to 250 °C with a split injection mode for a split ratio of 50:1. Separation was performed on two columns with different polarity, non-polar DB-5MS (column 1) and polar DB-WAX (column 2) capillary columns (Agilent J&W Scientific, Folsom, CA, USA) with the same dimensions of 30 m × 0.25 mm i.d. × 0.25 µm film thickness. The oven temperature program was as follows: (1) Column 1: the initial temperature was 45 °C (held for 2 min); it then increased to 130 °C at a rate of 2 °C/min (held for 10 min), to 150 °C at a rate of 2 °C/min, and finally to 250 °C at a rate of 2 °C/min and isothermal for 10 min at 280 °C with a total experiment time of 70 min; (2) Column 2: the initial temperature was 40 °C (held for 4 min); it then increased to 200 °C at a rate of 3 °C/min, and to 240 °C at a rate of 20 °C/min. Triplicate injections were made for each sample.

Mass spectra were recorded at 70 eV at a scan mode from *m*/*z* 35 to 500. The transfer line temperature was 260 °C. The ion source and quadrupole temperatures were 230 °C and 130 °C, respectively. Data acquisition was performed with Agilent MSD Chemstation (F.01.03.2357). 

Compound identification involved the comparison of the mass spectra with the databases (Wiley and the National Institute of Standards and Technology (NIST) using a probability-based matching algorithm. Further identification was based on the relative retention indices compared with the literature [38] and the reference standards purchased from commercial sources or isolated in-house. 

The raw percentage from the peak area of each compound was obtained in full-scan GC/MS analyses (DB-5MS and DB-WAX columns). Further standardization was not carried out, since our aim was focused on identifying the essential oil compounds for species differentiation. 

### 3.5. Antimalarial Activity

The antimalarial activity of EOs from *Baccharis* species was determined using a colorimetric assay based on plasmodial lactate dehydrogenase (LDH) activity as described by Kumar et al. [53]. A suspension of red blood cells infected with D6 or W2 strains of *P. falciparum* was added to the wells of a 96-well plate containing test samples diluted in medium at several concentrations. Parasitic LDH activity was determined according to the method described by Makler and Hinrichs [54]. Chloroquine and artemisinin were included as the drug controls. IC_50_ values were calculated from the dose-response curves using Excelfit^®^. DMSO (0.25%) was used as the vehicle control. For calculating the selectivity index of the antimalarial activity of EOs, their toxicity to Vero cells (monkey kidney fibroblasts) was also determined. Essential oils at different concentrations were added, and plates were again incubated for 48 h. The number of viable cells was determined using a vital dye (WST-8). Doxorubicin was used as a positive control. 

### 3.6. Antitrypanosomal Activity

The screening that was employed to test the antitrypanosomal activity of the EOs of *Baccharis* species against *T. brucei* was detailed in a previous paper by Jain et al. [55]. Briefly, the samples were tested against trypomastigotes cultures of *T. brucei*. The cell cultures of *T. brucei* were treated with varying concentrations of the samples, and the growth of the parasite cells were monitored with Alamar blue assay. The results were analyzed with ExcelFit^®^ to determine the IC_50_ and IC_90_ values.

### 3.7. Insecticidal Studies against Bed Bugs

The bed bug strains (Bayonne ‘Insecticide resistant’ and Ft. Dix ‘Susceptible’) were provided by Dr. Changlu Wang, Department of Entomology, Rutgers University, New Brunswick, NJ, and their colony was raised as explained by Montes et al. [56] using blood feeders (CG-1836-75 ChemGlass). The insecticidal activity of EOs against bed bugs was evaluated by fumigation, topical application, and residual studies. For fumigation test, the bed bugs were subjected to vapor toxicity in 125-mL clear glass jars using two microliter aliquots of 125 μg/μL EO stock solution that was injected directly onto inner bottle wall ~4 cm from the bottom. The jars were covered immediately with a screw cap and then sealed with parafilm ‘M’. The jars were then placed in the growth chamber, and data for mortality was recorded 24 h after treatment. Solutions were made in acetone, and the control treatment received acetone only. 2,2-Diclorovinil-dimetilfosfato (DDVP) was used as the standard.

Studies in topical application were performed with adult bugs, which were separated in the Petri dishes and anesthetized with CO_2_. Using a hand-held repeating dispenser, 1 μL of treatment solution (50 µg/bug) in acetone was delivered onto the dorsal surface of the abdomen. Control bugs received 1 μL of acetone alone. Data for the mortality of the bed bugs was recorded for seven days after treatment. There were three replicates with 10 bugs (mixed sex)/replicate. Deltamethrin was used as the standard (2.4 ng/bug). 

For residual studies, the method described by Campbell and Miller [57] was used with minor modifications. A 100-µL aliquot of treatment (diluted in acetone) was applied on 20-cm^2^ Whatman #1 filter paper achieving 100 μg/cm^2^ of residues. The treated filter papers were then placed in the Petri dish. Control treatments received only acetone. Ten adult bugs were released on the filter paper and mortality was recorded as mentioned in topical application. Deltamethrin was used as standard. Data of insecticidal investigations were analyzed for means, standard error, and one-way ANOVA in JMP 10.0.

### 3.8. Microscopic Procedure

The methods employed for light and scanning electron microscopy analysis of leaves and stems of *Baccharis* species are fully detailed in a previous paper by Budel et al. [4].

### 3.9. DNA Extraction, PCR, Sequencing

To extract genomic DNA from *Baccharis*, 100 mg of freeze-dried leaves were ground to fine powder. Genomic DNA from *Baccharis* samples was extracted using the DNeasy Plant Mini Kit (Qiagen Inc., Valencia, Spain). Four genomic regions—namely ITS, ETS, *psb*A-*trn*H, and *trn*L-*trn*F—were amplified in 25-µL reactions.

The PCR consisted of a 25-μL reaction mixture containing 2 μL of the DNA solution, 1x PCR reaction buffer, 0.2 mM of dNTP mixture, 0.2 μM of each forward and reverse primers (Table 5), 1.5 mM of MgCl_2_ and 1 U of Platinum Taq DNA Polymerase (Invitrogen, Carlsbad, CA, USA). The program comprised of one initial denaturation step at 94 °C for 3 min, followed by 35 cycles at 94 °C for 30 s, X °C for 30 s, and 72 °C for X s (see Table 5 for annealing temperature and extension time). 

After amplification, each PCR reaction was analyzed by electrophoresis on a 1.5% borate agarose gel and visualized under UV light. The sizes of the PCR products were compared to the molecular size standard 1 kb plus DNA ladder (cat no.: 10787-018, Invitrogen, Carlsbad, CA, USA).

Successfully amplified PCR products were isolated with NucleoSpin^®^ Gel and a PCR Clean-up kit (MACHEREY-NAGEL, cat no. 740609.50) and eluted with 30 μL of Buffer AE from the DNeasy Plant Mini Kit (Qiagen Inc., Valencia, Spain). PCR products were sequenced in both directions at GeneWiz (South Plainfield, NJ, USA). Sequences were analyzed with DNASTAR (DNASTAR, Madison, WI, USA) and Clone manager 9 (Scientific and Educational Software, Cary, NC, USA) and visually inspected. Contiguous sequences were screened against previously sequenced authenticated samples.

## 4. Conclusions

In the present work, profiles of EOs from five species of *Baccharis* were analyzed and compared. The chemical compositions of EOs of *B. pauciflosculosa*, *B. reticularioides*, and *B. sphenophylla* are reported for the first time. Although the qualitative compositions of the EOs of these species were more or less similar, they showed distinctive differences in the quantity of the components. Some compounds were unique to these species, and hence can be used as chemical markers for species identification and authentication. *B. microdonta* differed from the other species by having kongol and spathulenol in high concentrations. *B. pauciflosculosa* showed β-pinene and limonene as major compounds. α-Bisabolol was found only in *B. punctulata*. *B. reticularioides* showed α-pinene, while *B. sphenophylla* presented α-pinene, β-pinene, limonene, and spathulenol as major compounds. 

*B. microdonta* and *B. punctulata* exhibited cytotoxicity, whereas *B. pauciflosculosa*, *B. reticularioides*, and *B. sphenophylla* showed moderate antimalarial activities. Only *B. sphenophylla* EO showed strong toxicity to bed bug *viz*., and 66.67% and 83.33% mortality in ‘Bayonne’ and ‘Ft.Dix’ in the fumigation bioassay. *B. pauciflosculosa* and *B. reticularioides* showed good antitrypanosomal activities. 

The leaves and stems of all five *Baccharis* species possessed glandular trichomes and ducts as secretory structures. All three types of glandular trichomes that were observed in this study contained EOs. DNA barcoding using ITS and trnL-trnF sequences were useful for the authentication of the studied *Baccharis* species.

## Figures and Tables

**Table 1:** Chemical compositions of the essential oils of *Baccharis* species. RRI: relative retention indices.

No.	RRI ^a^	RI Lit ^b^	RRI ^c^	RI Lit ^d^	Compound Name	Peak Area % ^e^										
						*B. microdonta*		*B. pauciflosculosa*		*B. punctulata*		*B. reticularioides*		*B. sphenophylla*		ID
						NC	PC	NC	PC	NC	PC	NC	PC	NC	PC	
1	937	924	1019	1038	α-Thujene	-	-	3.42	2.94	0.41	0.43	0.89	1.18	0.54	0.37	MS, RI
2	942	932	1014	1036	**α-Pinene**	0.72	0.73	**10.45**	**9.44**	3.55	3.15	**24.50**	**24.78**	**10.74**	8.04	t*_R_*, MS, RI
3	954	946	1050	1083	Camphene ^f^	-	-	0.78	0.75	0.19	0.18	0.43	0.42	0.39	0.30	t*_R_*, MS, RI
4	950	953	1109	-	Thuja-2,4(10)-diene	-	-	-	-	-	-	2.91	3.65	0.12	0.09	MS, RI
5	973	969	1105	1130	Sabinene ^f^	-	-	2.75	2.62	0.89	0.79	0.39	0.85	3.82	3.18	t*_R_*, MS, RI
6	976	974	1089	1124	**β-Pinene**	2.24	2.33	**18.33**	**16.50**	4.95	4.41	7.68	9.24	**15.24**	**13.17**	t*_R_*, MS, RI
7	988	988	1156	1156	β-Myrcene	-	-	3.65	2.76	0.30	0.23	0.27	0.29	0.67	0.57	t*_R_*, MS, RI
8	1001	1002	1151	1177	α-Phellandrene	-	-	-	-	0.33	0.11	-	-	-	-	t*_R_*, MS, RI
9	1003	1008	-	1141	δ-(3)-Carene	-	-	-	-	-	-	-	-	0.99	0.82	t*_R_*, MS, RI
10	1010	1014	1198	1188	α-Terpinene	-	-	0.19	-	0.10	-	0.43	0.39	0.58	0.64	t*_R_*, MS, RI
11	1018	1020	1271	1272	*p*-Cymene	-	-	0.82	1.24	3.44	1.94	3.18	3.27	1.31	1.79	t*_R_*, MS, RI
12	1021	1024	1189	1206	**Limonene ^f^**	1.12	1.14	**18.77**	**14.99**	**11.35**	**9.77**	2.47	2.75	**14.33**	**11.81**	t*_R_*, MS, RI
13	1040	1044	1254	1250	*trans-β-*Ocimene	-	-	-	-	0.25	0.14	0.14	0.08	-	-	MS, RI
14	1049	1054	1243	1251	γ-Terpinene	-	-	0.31	-	0.16	0.06	0.70	0.38	1.03	0.41	t*_R_*, MS, RI
15	1074	1086	1267	1287	Terpinolene ^f^	-	-	-	-	-	-	0.44	0.20	0.37	0.18	t*_R_*, MS, RI
16	1079	1089	1462	-	*p*-Cymenene	-	-	-	-	-	-	0.85	1.01	0.11	0.12	MS, RI
17	1092	1095	1593	1506	Linalool ^f^	0.14	-	0.41	0.57	0.31	0.37	-	-	0.15	0.22	t*_R_*, MS, RI
18	1106	1101	1449	-	Thujone ^f^	-	-	-	-	-	-	0.52	0.55	-	-	t*_R_*, MS, RI
19	1115	1122	1518	-	α-Campholenal	-	-	-	-	-	-	1.63	2.38	-	-	MS, RI
20	1124	1135	1607	-	Nopinone	-	-	-	-	-	-	0.38	0.42	-	-	MS, RI
21	1127	1135	1695	-	*trans*-Pinocarveol	0.73	1.23	0.41	0.92	0.27	0.37	4.44	6.84	0.79	1.23	t*_R_*, MS, RI
22	1133	1140	1700	-	*trans*-Verbenol	-	-	0.10	0.34	0.11	0.18	0.94	0.61	0.11	-	MS, RI
23	1138	-	1706	-	Unknown 1 [*m*/*z* 94 (100%), 79 (89.9%), 59 (79.7%), 91 (51.7%)]	-	-	-	-	-	-	2.12	2.10	-	-	MS
24	1148	1160	1597	-	Pinocarvone ^f^	0.32	0.29	0.26	0.16	0.16	0.09	0.80	1.03	0.27	0.34	t*_R_*, MS, RI
25	1161	-	1769	-	α-Phellandren-8-ol	-	-	-	-	-	-	5.60	3.72	0.15	0.03	MS
26	1168	1174	1643	1628	Terpinene-4-ol ^f^	-	-	1.19	1.23	0.47	0.44	1.34	1.29	3.10	3.45	t*_R_*, MS, RI
27	1176	1179	1882	1846	*p*-Cymene-8-ol	-	-	0.11	0.17	-	-	0.83	1.16	0.21	0.32	MS, RI
28	1181	1195	1659	-	Myrtenal ^f^	0.59	0.67	0.34	0.41	0.24	0.27	2.14	3.25	0.53	0.62	t*_R_*, MS, RI
29	1184	1186	1740	1731	α-Terpineol	0.97	0.65	0.92	0.74	0.42	0.26	4.82	0.61	1.85	1.42	t*_R_*, MS, RI
30	1193	1204	1737	1733	Verbenone ^f^	-	-	-	-	-	-	2.95	2.65	-	-	t*_R_*, MS, RI
31	1208	1225	1850	1820	*cis*-Carveol	0.15	-	0.13	0.34	0.27	0.30	1.19	1.15	0.27	0.38	MS, RI
32	1231	1239	1765	1715	Carvone ^f^	0.17	-	0.13	0.22	0.42	0.46	0.43	0.46	0.22	0.31	MS, RI
33	1235	-	1867	-	2-Carene-4-ol	-	-	-	-	-	-	0.32	0.17	-	-	MS
34	1271	1284	1615	1599	Bornyl acetate ^f^	-	-	-	-	1.32	1.04	0.60	0.51	0.12	0.09	t*_R_*, MS, RI
35	1356	1374	1515	1493	α-Copaene	0.26	0.28	0.21	0.33	-	-	-	-	0.26	0.34	MS, RI
36	1370	1389	1621	1591	β-Elemene	0.87	1.10	0.41	0.51	0.67	0.53	-	-	0.23	-	MS, RI
37	1395	1417	1624	1617	β-Caryophyllene	0.97	0.92	1.80	1.55	3.35	2.76	-	-	3.61	3.46	t*_R_*, MS, RI
38	1427	1437	1707	1672	α-Humulene	0.39	0.26	0.19	-	0.42	0.41	-	-	0.31	0.12	t*_R_*, MS, RI
39	1447	1478	1720	1692	γ-Muurolene	0.15	0.28	0.26	0.56	0.17	0.54	-	-	0.27	0.47	MS, RI
40	1451	1484	1737	1712	Germacrene-D	0.64	0.41	2.56	0.68	3.63	2.48	-	-	1.44	0.80	MS, RI
41	1458	1489	1746	1756	β-Selinene	1.35	1.23	0.15	0.11	0.12	0.16	0.18	0.07	0.24	0.21	MS, RI
42	1460	1496	1725	-	Ledene^f^	-	-	0.29	0.10	0.12	0.13	-	-	0.17	0.14	t*_R_*, MS, RI
43	1464	1500	1761	1744	Bicyclogermacrene ^f^	-	-	1.25	0.01	3.10	2.85	-	-	0.48	0.05	MS, RI
44	1464	1498	1751	1729	α-Selinene	0.58	0.48	-	-	-		-		-		MS, RI
45	1469	1500	1756	1730	α-Muurolene	0.14	-	0.63	0.73	0.26	0.27	-	-	0.29	0.26	MS, RI
46	1480	1505	1705	1745	β-Bisabolene	-	-	-	-	1.18	0.78	-	-	-	-	MS, RI
47	1488	1522	1787	1761	δ-Cadinene	1.00	1.17	2.74	1.96	1.13	1.35	0.28	1.04	0.79	1.52	MS, RI
48	1509	1544	1930	1916	α-Calacorene	0.78	0.37	-	-	-	-	0.43	0.18	0.18	0.21	MS, RI
49	1520	1548	2080	2078	Elemol ^f^	-	-	0.12	-	1.02	1.09	-	-	-	-	MS, RI
50	1537	1561	2051	2044	(*E*)-Nerolidol	-	-	0.43	0.60	0.13	0.30	-	-	0.17	0.17	MS, RI
51	1540	1567	1942	1931	Palustrol ^f^	3.23	3.22	-	-	0.13	-	-	-	-	-	MS, RI
52	1551	1577	2114	2153	**Spathulenol ^f^**	**22.74**	**24.19**	9.53	**12.18**	9.96	**11.66**	5.52	2.70	**13.15**	**14.92**	t*_R_*, MS, RI
53	1555	1582	1987	1966	Caryophyllene oxide ^f^	6.84	7.47	2.11	3.44	5.30	6.01	1.37	1.34	5.34	6.78	t*_R_*, MS, RI
54	1559	1590	2128	-	Globulol	0.59	0.69	-	-	-	-	-	-	0.36	0.32	MS, RI
55	1571	1592	2080	2112	Viridiflorol ^f^	4.36	4.90	1.81	2.69	-	-	-	-	1.66	2.01	t*_R_*, MS, RI
56	1585	1602	2031	-	Ledol ^f^	2.38	2.55	-	-	-	-	-	-	-	-	MS, RI
57	1622	1627	2063	2037	1-*epi*-Cubenol	1.03	0.57	0.38	0.39	-	-	-	-	-	-	MS, RI
58	1625	1630	-	-	γ-Eudesmol	-	-	-	-	1.09	1.65	-	-	-	-	MS, RI
59	1630	-	-	-	Unknown 2 [*m*/*z* 119 (100%), 105 (92.9%), 91 (90.2%), 93 (79.5%)]	1.37	1.22	-	-	-	-	-	-	-	-	MS
60	1633	1638	2094	-	*epi*-α-Cadinol	0.18	0.38	-	-	1.95	2.09	-	-	-	-	MS, RI
61	1644	1644	2151	2150	δ-Cadinol	0.42	0.33	0.77	0.65	0.26	0.64	2.64	1.56	2.27	2.03	MS, RI
62	1647	1640	2164	-	*epi*-α-Muurolol	0.19	-	0.65	0.75	0.27	0.33	-	-	-	-	MS, RI
63	1659	1649	2196	2248	β-Eudesmol	-	-	-	-	0.64	1.65	-	-	-	-	t*_R_*, MS, RI
64	1661	1656	2120	-	α-Bisabolol oxide B	-	-	-	-	1.17	0.47	-	-	-	-	MS, RI
65	1662	1652	2200	2224	α-Cadinol	-	-	1.44	2.08	-	-	1.36	0.46	1.49	2.10	MS, RI
66	1665	-	2214	-	**Kongol**	**22.22**	**20.09**	-	-	-	-	-	-	-	-	t*_R_*, MS
67	1672	1675	2209	2203	Cadalene	-	-	-	-	1.34	1.32	-	-	-	-	MS, RI
68	1697	-	2278	-	Murolan-3,9(11)-diene-10-peroxy	0.54	0.64	-	-	-	-	-	-	-	-	MS
69	1705	-	2292	-	(1*R*,7*S*,*E*)-7-Isopropyl-4,10-dimethylenecyclodec-5-enol	0.83	0.94	0.39	0.28	-	-	-	-	0.42	0.46	MS
70	1708	1685	2190	2022	**α-Bisabolol**	-	-	-	-	**23.63**	**20.72**	-	-	-	-	t*_R_*, MS, RI
Compounds identified (%)						58.33	52.63	77.36	67.27	77.96	73.33	74.50	66.66	76.66	69.84	
Monoterpenoids hydrocarbons						6.67	5.26	20.75	16.36	22.03	20.00	27.45	24.56	25.00	23.81	
Oxygenated monoterpenoids						10.00	7.02	16.98	16.36	15.26	15.00	33.33	29.82	18.33	15.87	
Sesquiterpenoids hydrocarbons						18.33	17.54	20.75	18.19	18.64	18.33	5.88	5.26	20.00	17.46	
Oxygenated sesquiterpenoids						23.33	22.81	18.88	16.36	22.03	20.00	7.84	7.02	13.33	12.70	

RRI ^a^, relative retention indices calculated against n-alkanes on the DB-5MS column; RI lit ^b^, retention index literature (DB-5 column) [37]; RRI ^c^, relative retention indices calculated against n-alkanes on the DB-WAX column; RI lit ^d^, retention index literature (CW20M column) [38], Peak Area% ^e^; stereoisomers not identified ^f^; NC, non-polar column; PC, polar column, t*_R_*, identification based on the retention times (t*_R_*) of genuine compounds on the DB-5MS column; MS, identified on the basis of computer matching of the mass spectra with those of the Wiley and NIST libraries and comparison with literature data. The compounds in bold represent the major compounds.

**Table 2:** Activities of essential oils of *Baccharis* species against *Plasmodium falciparum*.

Sample Name	*P. falciparum* (D6 Clone)		*P. falciparum* (W2 Clone)		Cytotoxicity (Vero Cells) IC_50_ (µg/mL)
	IC_50_ (µg/mL)	SI	IC_50_ (µg/mL)	SI	
*B. microdonta*	14.75 ± 3.80	2.4	23.93 ± 4.64	1.5	35.80 ± 7.29
*B. pauciflosculosa*	10.90 ± 0.98	>4.3	14.20 ± 1.08	>3.3	NC
*B. punctulata*	17.26 ± 0.83	2.2	19.73 ± 4.11	1.9	37.81 ± 6.36
*B. reticularioides*	20.32 ± 4.37	>2.3	34.35 ± 10.15	>1.4	NC
*B. sphenophylla*	27.58 ± 1.64	>1.7	32.53 ± 16.5	>1.5	NC
Chloroquine	0.014	>17	0.117	>2	NC
Artemisinin	0.004	>31.8	0.003	>71.3	NC

NC: No cytotoxicity up to 47.6 μg/mL of essential oils and 0.238 µg/mL for chloroquine and artemisinin; SI: selectivity index (IC_50_ for cytotoxicity/IC_50_ for antimalarial activity); values were measured in triplicate (*n* = 3). They are presented as mean ± SD.

**Table 3:** In vitro antitrypanosomal activity of essential oils of *Baccharis* species against *T. brucei.*

Sample Name	IC_50_ (µg/mL) *	IC_90_ (µg/mL) *
*B. microdonta*	1.688 ± 0.354	2.683 ± 0.123
*B. pauciflosculosa*	0.306 ± 0.056	0.516 ± 0.043
*B. punctulata*	1.054 ± 0.211	1.969 ± 0.201
*B. reticularioides*	0.955 ± 0.121	2.484 ± 0.165
*B. sphenophylla*	1.143 ± 0.113	2.378 ± 0.201
Pentamidine	0.007 ± 0.001	0.011 ± 0.002
α-Difluoromethylornithine (DFMO)	5.506 ± 0.412	12.052 ± 0.613

* Values were measured in triplicate (*n* = 3). They are presented as mean ± SD.

**Table 4:** KP2 distances between five *Baccharis* samples and authenticated species.

	Lowest Kp Distance (ITS)		Lowest Kp Distance (trnL-trnF)		Lowest Kp Distance (ETS)		Lowest Kp Distance (psbA-trnH)				
Sample	Species Match	Value	Species Match	Value	Species Match	Value	Species Match(es)				Value
B. pa.	B. pa	0.000	B. il	0.000	B. pa	0.003	B. pa	B. il	B. re	B. sp	0.004
B. re	B. re	0.000	B. il	0.001	B. re	0.001	B. pa	B. il	B. re		0.000
B. mi	B. mi	0.001	B. mi	0.000	NA		B. mi				0.000
B. pu	B. pu	0.006	B. pu	0.000	NA		B. pu				0.000
B. sp	B. sp	0.000	B. il	0.004	NA		B. pa	B. il	B. re		0.000

A KP2 distance of 0.000 represents a 100% identity match of a sample with a species. Authenticated samples B. mi: *B. microdonta* (GH1599), B. pa: *B. pauciflosculosa* (GH1558), B. il: *B. illinita* (GH1586), B. pu: *B. punctulata* (GH1892), B. re: *B. reticularioides* (GH1426), B. sp: *B. sphenophylla* (GH1438), NA: not analyzed due to low sequence quality.

**Table 5:** List of primers, T_M_, and extension time.

Genomic Regions	Sequence in 5′-3′	Source	T_M_	Extension Time at 72 °C
**ETS1f**	CTTTTTGTGCATAATGTATATATAGGGGG	Linder et al. [58]	45 °C	60 s
**18S-2L**	TGACTACTGGCAGGATCAACCAG			
**ITS4**	TCCTCCGCTTATTGATATGC	White et al. [59]	52 °C	30 s
**ITS5**	GGAAGTAAAAGTCGTAACAAGG			
**trnL-F-trnC**	CGAAATCGGTAGACGCTACG	Taberlet et al. [60]	52 °C	60 s
**trnL-F-trnF**	ATTTGAACTGGTGACACGAG			
**psbA**	CGAAGCTCCATCTACAAATGG	Hamilton et al. [61]	56 °C	30 s
**trnH (GUG)**	ACTGCCTTGATCCACTTGGC			

T_M_ = Melting temperature.

## Supplementary Materials

The supplementary materials are available online, Figure S1: Proton and Carbon NMR spectra of kongol, Figure S2: Proton and Carbon NMR spectra of spathulenol.
