# Supplementary material for: Essential Oils of Five Baccharis Species: Investigations on the Chemical Composition and Biological Activities
Source: Molecules. 2018 Oct 12;23(10):2620. doi: 10.3390/molecules23102620 (PMC6222634; doi:10.3390/molecules23102620)
Supplement: Supplementary file 1 [file molecules-23-02620-s001.pdf]

molecules

MDPI

Article

Essential oils of five *Baccharis* species: investigations on the chemical composition and biological activities

Jane M. Budel [1,2]*, Mei Wang [2], Vijayasankar Raman [2], Jianping Zhao [2], Shabana I. Khan [2], Junaid U. Rehman [2], Natascha Techen [2], Babu Tekwani [2], Luciane M. Monteiro [1], Gustavo Heiden [4], Inês J. M. Takeda [5], Paulo V. Farago [1] and Iklas A. Khan [2]

[1] Departamento de Ciências Farmacêuticas, Universidade Estadual de Ponta Grossa (UEPG), Ponta Grossa, PR, Brasil; janemanfron@hotmail.com; lmmonteiro@hotmail.com; pvfarago@gmail.com

[2] National Center for Natural Products Research, School of Pharmacy, University of Mississippi, University, MS, USA; meiwang@olemiss.edu; vraman@olemiss.edu; jianping@olemiss.edu; skhan@olemiss.edu; jurheman@olemiss.edu; ntechen@olemiss.edu; btekwani@olemiss.edu

[3] Embrapa Clima Temperado, Pelotas, RS, Brasil; gustavo.heiden@embrapa.br

[4] Departamento de Meio Ambiente, Universidade Estadual de Maringá (UEM), Umuarama, PR, Brasil; takedaines@bol.com.br

* Correspondence: janemanfron@hotmail.com; Tel.: +554133203000

Received: date; Accepted: date; Published: date

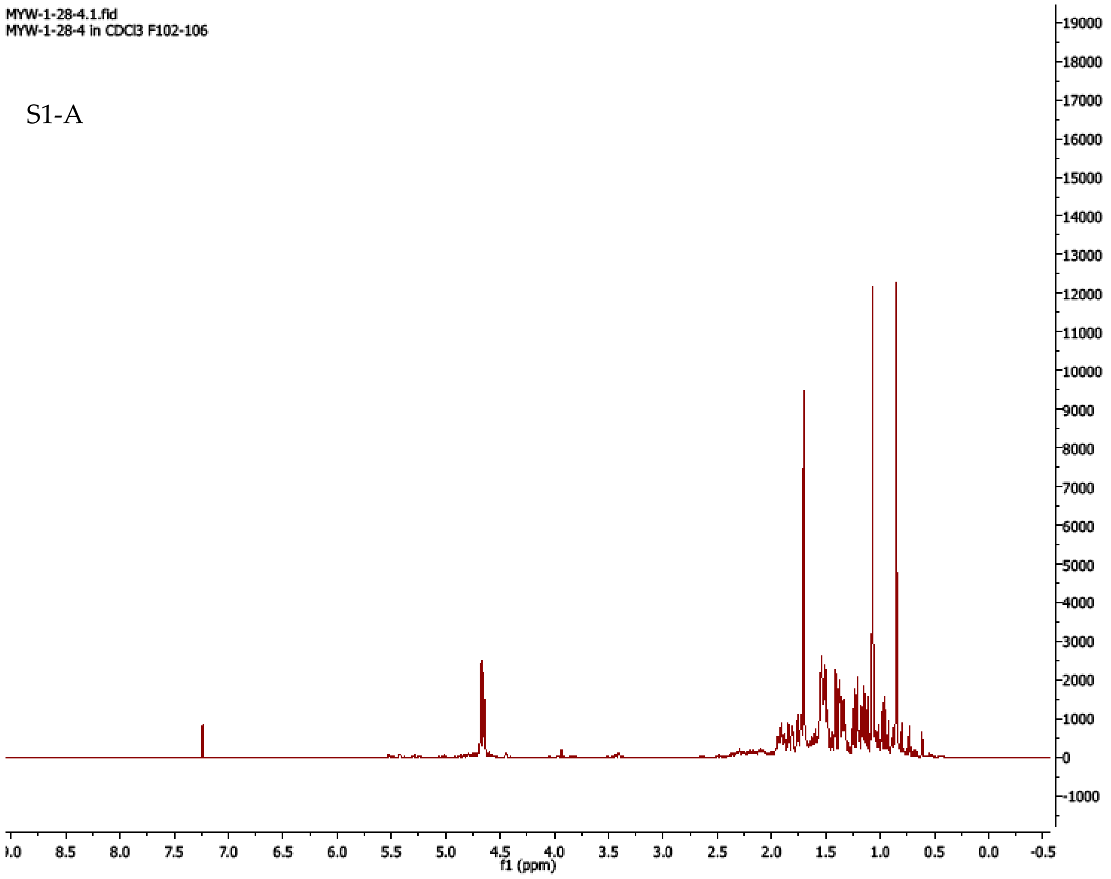

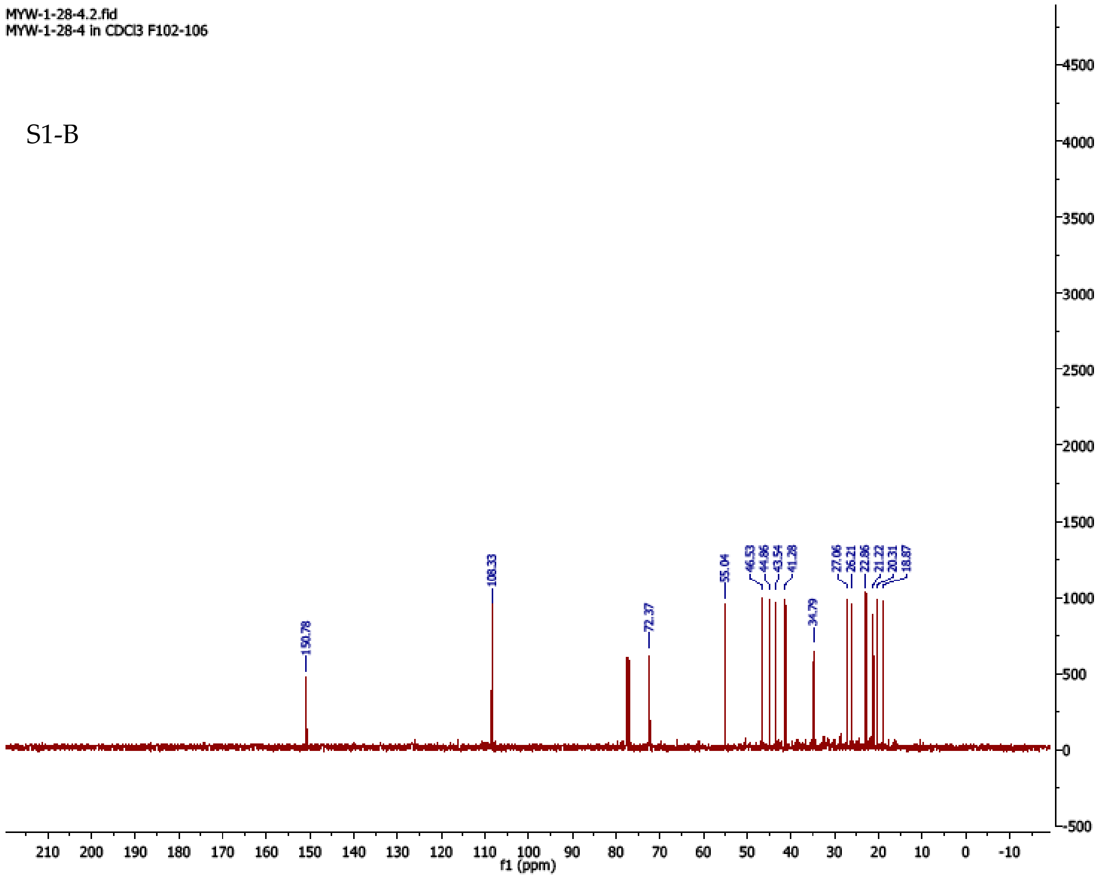

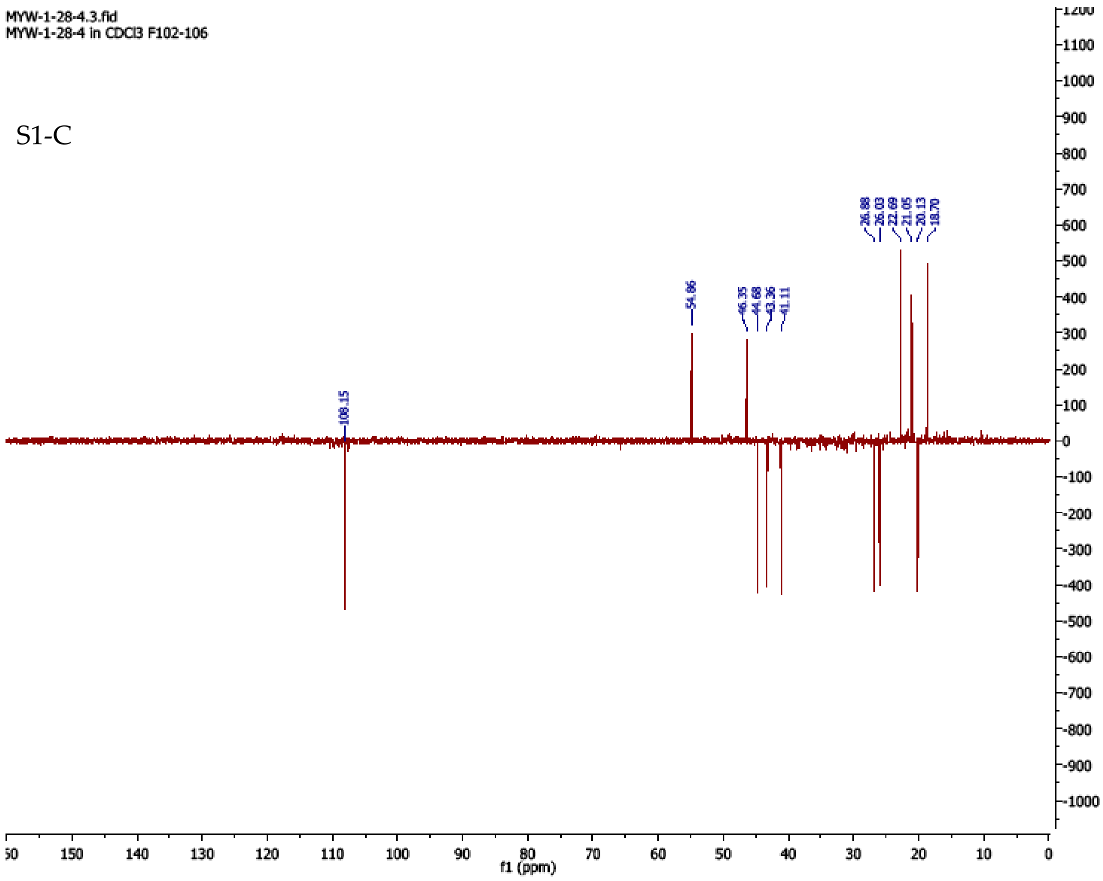

Figure S1. Proton and Carbon NMR spectra of kongol

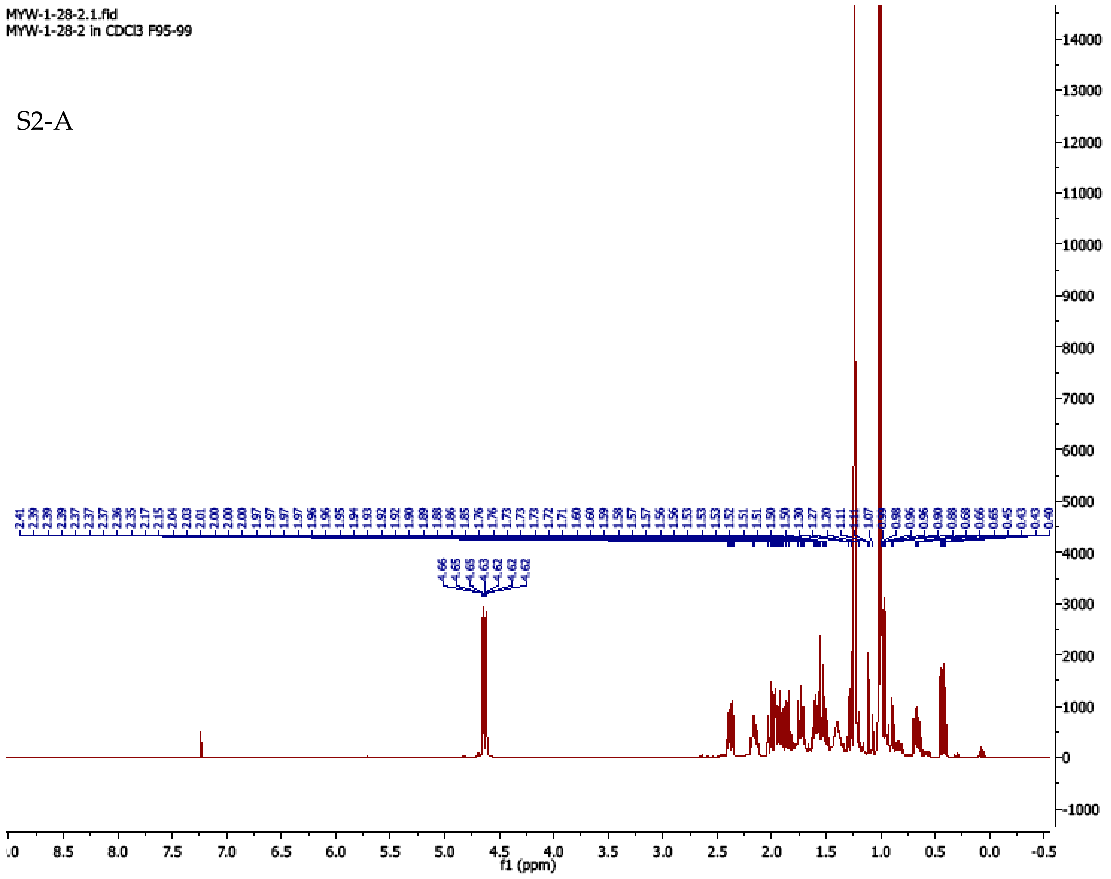
MYW-1-28-2.1.fid
MYW-1-28-2 in CDCl3 F95-99
S2-A
f1 (ppm)

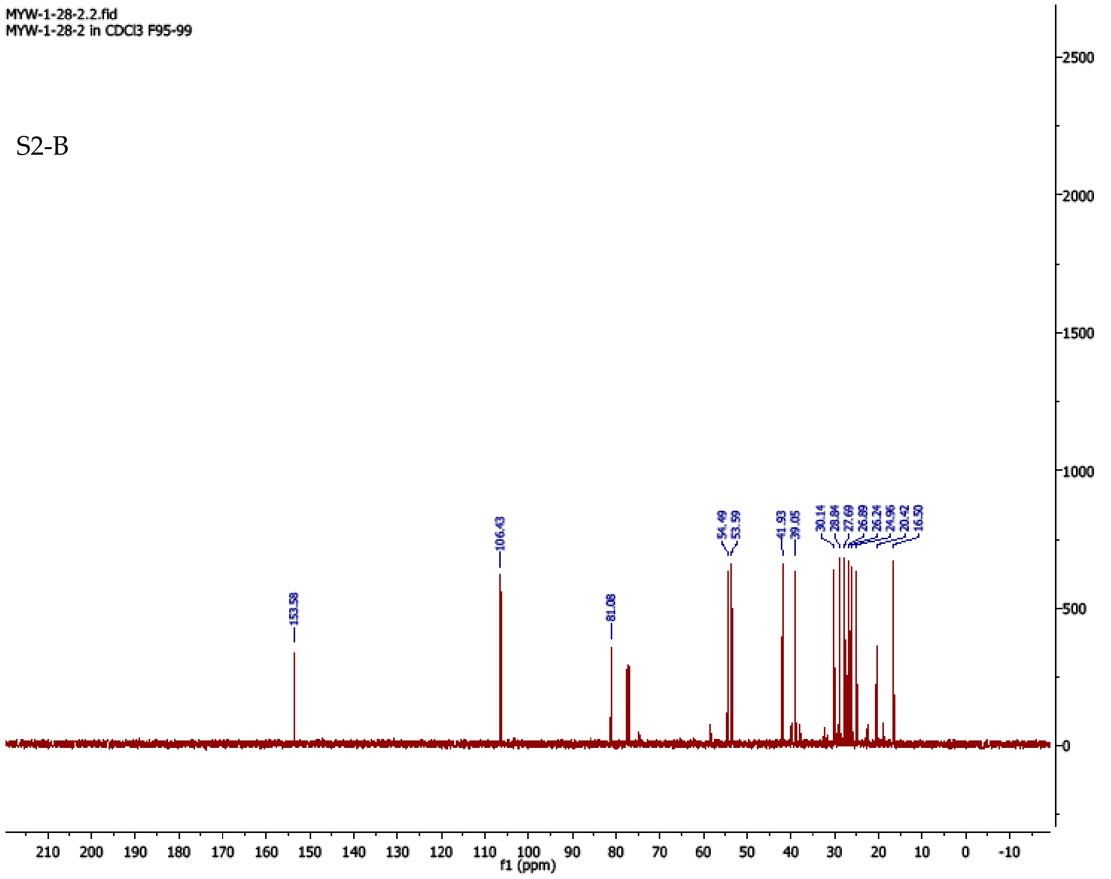
MYW-1-28-2.2.fid
MYW-1-28-2 in CDCl3 F95-99
S2-B
153.58
106.43
81.08
54.49
53.59
41.93
39.05
30.14
28.84
27.69
26.89
26.24
24.96
20.42
16.50
f1 (ppm)

S2-C

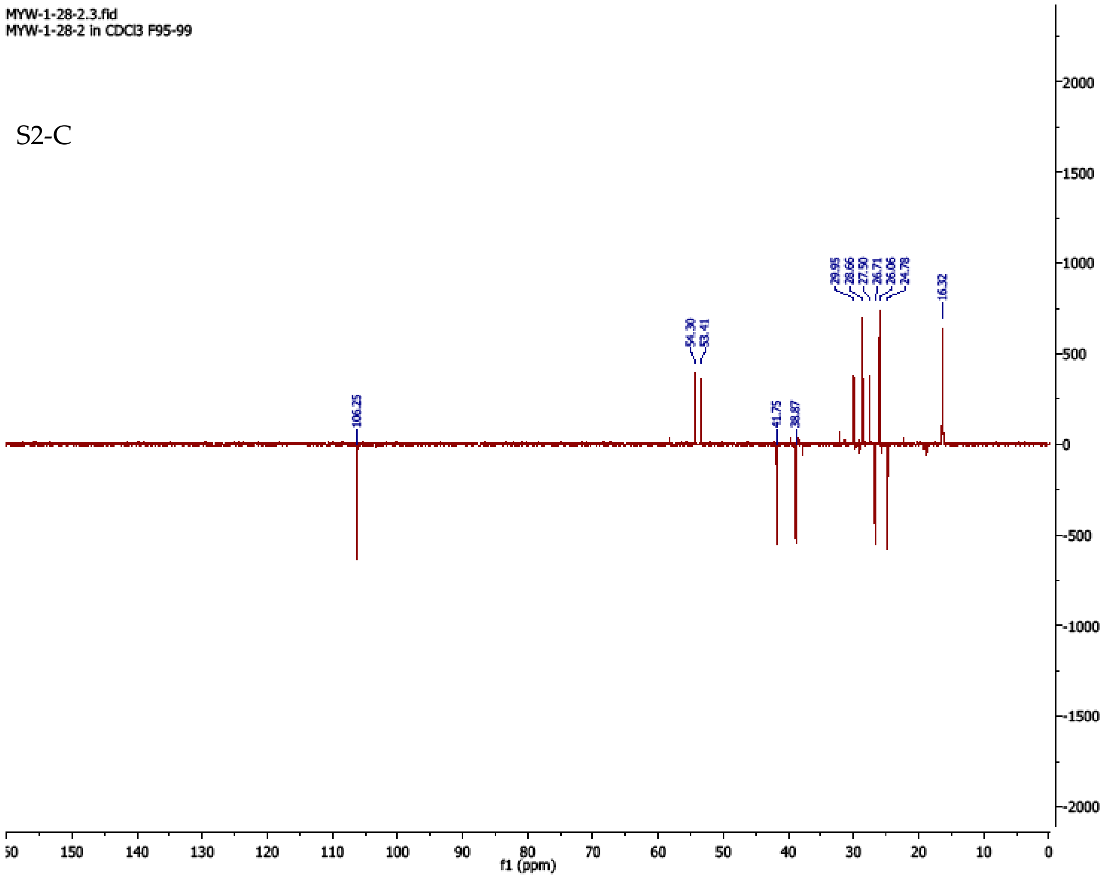

Figure S2. Proton and Carbon NMR spectra of spathulenol

© 2018 by the authors. Submitted for possible open access publication under the terms and conditions of the Creative Commons Attribution (CC BY) license (http://creativecommons.org/licenses/by/4.0/).
